# Patterns of User Engagement With the Mobile App, Manage My Pain: Results of a Data Mining Investigation

**DOI:** 10.2196/mhealth.7871

**Published:** 2017-07-12

**Authors:** Quazi Abidur Rahman, Tahir Janmohamed, Meysam Pirbaglou, Paul Ritvo, Jane M Heffernan, Hance Clarke, Joel Katz

**Affiliations:** ^1^ Centre for Disease Modelling Department of Mathematics and Statistics York University Toronto, ON Canada; ^2^ ManagingLife, Inc. Toronto, ON Canada; ^3^ School of Kinesiology & Health Science York University Toronto, ON Canada; ^4^ Department of Psychology York University Toronto, ON Canada; ^5^ Department of Anesthesia and Pain Management Toronto General Hospital Toronto, ON Canada

**Keywords:** chronic pain, mhealth, opioid use, data mining, cluster analysis, Manage My Pain, pain management, pain app

## Abstract

**Background:**

Pain is one of the most prevalent health-related concerns and is among the top 3 most common reasons for seeking medical help. Scientific publications of data collected from pain tracking and monitoring apps are important to help consumers and healthcare professionals select the right app for their use.

**Objective:**

The main objectives of this paper were to (1) discover user engagement patterns of the pain management app, Manage My Pain, using data mining methods; and (2) identify the association between several attributes characterizing individual users and their levels of engagement.

**Methods:**

User engagement was defined by 2 key features of the app: longevity (number of days between the first and last pain record) and number of records. Users were divided into 5 user engagement clusters employing the k-means clustering algorithm. Each cluster was characterized by 6 attributes: gender, age, number of pain conditions, number of medications, pain severity, and opioid use. Z tests and chi-square tests were used for analyzing categorical attributes. Effects of gender and cluster on numerical attributes were analyzed using 2-way analysis of variances (ANOVAs) followed up by pairwise comparisons using Tukey honest significant difference (HSD).

**Results:**

The clustering process produced 5 clusters representing different levels of user engagement. The proportion of males and females was significantly different in 4 of the 5 clusters (all *P* ≤.03). The proportion of males was higher than females in users with relatively high longevity. Mean ages of users in 2 clusters with high longevity were higher than users from other 3 clusters (all *P* <.001). Overall, males were significantly older than females (*P* <.001). Across clusters, females reported more pain conditions than males (all P <.001). Users from highly engaged clusters reported taking more medication than less engaged users (all *P* <.001). Females reported taking a greater number of medications than males (*P* =.04). In 4 of 5 clusters, the percentage of males taking an opioid was significantly greater (all *P* ≤.05) than that of females. The proportion of males with mild pain was significantly higher than that of females in 3 clusters (all *P* ≤.008).

**Conclusions:**

Although most users of the app reported being female, male users were more likely to be highly engaged in the app. Users in the most engaged clusters self-reported a higher number of pain conditions, a higher number of current medications, and a higher incidence of opioid usage. The high engagement by males in these clusters does not appear to be driven by pain severity which may, in part, be the case for females. Use of a mobile pain app may be relatively more attractive to highly-engaged males than highly-engaged females, and to those with relatively more complex chronic pain problems.

## Introduction

Internet-based and mobile health (mHealth) apps are transforming how people monitor, manage, and communicate health-related information [1]. This trend has been documented in the fields of medicine [2], nursing [3], psychology [4], kinesiology [5], nutrition [6], and for multiple health concerns and diseases [1].

Pain is one of the most prevalent health-related concerns and is among the top 3 most common reasons for seeking medical help [7]. Several recent reviews have highlighted the many commercially available pain-related apps that can be downloaded from online app stores by people with chronic pain [8-11]. As of 2015, between 279 [8] and 283 [9] pain-related apps were commercially available to monitor and track pain. The rapid proliferation of mobile apps, in general, and for pain in particular, has not been accompanied by equal attention to determining the factors consumers and healthcare professionals prefer or require when selecting from among the many available apps. App quality, usability, effectiveness, and other relevant data for most mHealth apps are either unavailable, incomplete, or potentially inaccurate [1,8,9]. Consumers and healthcare providers have little reliable information to consult when seeking the best app for their needs. To illustrate the mismatch between pain-related app availability and reliable scientific data, de la Vega and Miró [9] noted that of the 34 pain-related apps evaluated in the published scientific literature, not one was available on any major online app store. Conversely, of the 283 pain-related apps commercially available at the major app stores, not one has been evaluated in a scientific publication.

Accordingly, the present study had 2 objectives. The first was to describe a first-of-a-kind collaboration between the award-winning mobile app Manage My Pain (developed to monitor and track pain) and pain, mental health, and data mining experts. The second objective was to present data from greater than 24,000 users (comprising more than 544,000 data points) by clustering data using key variables that defined the user base. Specifically, using a measure of user engagement with the app (eg, what distinguishes the user who has used the app frequently and over the longer term from others?), defined by the longevity and number of records for each user, we were able to group the users (using clustering methods) into 5 groups differentiated by high or low number of entries and high or low longevity. We then characterized the 5 groups of users by gender, as well as other attributes collected by the app: age, number of pain conditions, number of current medications, opioid use, and pain severity rating.

## Methods

### Manage My Pain

Manage My Pain [12], developed by ManagingLife, helps people living with pain to track their pain and functioning daily basis using an Android mobile phone app. Since Manage My Pain was launched in 2011, more than 24,000 people have created an account and recorded their pain. In total, more than 544,000 pain episodes have been documented by users.

**Figure 1:**
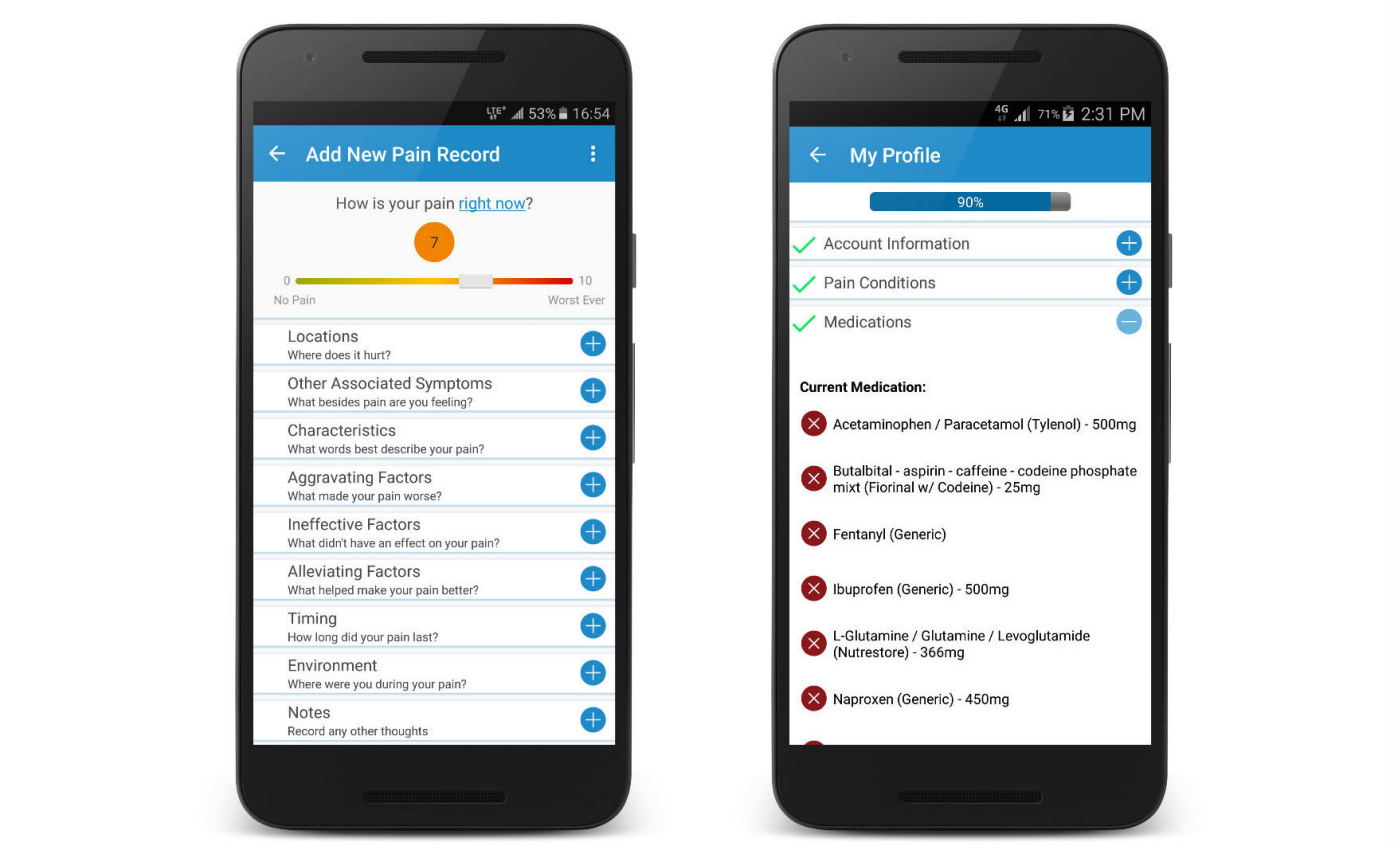
Screenshots of Manage My Pain showing how pain episodes are recorded (left) and where users can capture information about themselves (right).

The central feature of Manage My Pain is the “pain record” that enables users to enter details about their pain episodes. Users are asked to complete only 1 item, a rating of pain severity using a slider on a visual analogue scale. They then have the option of completing 7 more items regarding their present pain that typically take less than 1 minute to complete (Figure 1). The app issues daily reminders and prompts users to reflect on their daily accomplishments. With regular use, users are empowered and gain self-awareness through charts and graphs that provide insight to their pain and functioning and how it changes over time.

The information collected by the app can be summarized into a report intended for clinical use, where the information collected is presented in a concise fashion and primarily focuses on changes in the self-reported outcome data between clinical visits. Output is structured on a single page and tends to be more accurate than a patient’s recollection of pain since the last clinical visit, as it captures pain closer to the time of experience and is less influenced by recency and recall biases that plague existing methods for capturing pain information [13]. To supplement the information presented in the reports, users can add pain conditions, gender, age, and medications to their profile in the app.

The app supports 7 languages (English, Spanish, French, German, Russian, Simplified Chinese, and Korean) and has users from over 130 countries. It is available free in a Lite version, or users can opt to pay a one-time fee for a Pro version. The only difference between the versions is that the former limits the number of records that can be viewed at one time to 10. If users choose to take advantage of ManagingLife’s secure, cloud-based storage, they can create an online account and agree to ManagingLife’s Privacy Policy [14], which includes consent to use their aggregated and de-identified data for research purposes. Creating an account not only enables cross-device synchronization through encrypted data transmission and secure storage in the instance a device is lost, it also enables features such as advanced report generation and access to the Profile section of the app (Figure 1). The majority of the analysis in this paper is derived from the self-reported information contained in the Profile section of the app. Users also have the ability to use Manage My Pain without creating an account in which case data does not leave the device and are therefore not accessible for research such as the present report.

### Procedure

The present study was reviewed and approved by the Research Ethics Board at York University (Human Participants Review Committee, Certificate e2015-160). The users’ database was accessed and downloaded in 2 separate files (plain text format): user information and pain records. The user-information file contained the field's user identification (ID), date of birth, gender, pain conditions, and medications. Information specific to individual pain conditions included in the pain records file were date, location, other associated symptoms, characteristics, alleviating factors, ineffective factors, aggravating factors, severity, environment, pain type, and pain duration. All fields in the text files were delimited using special characters. The files used in this study were downloaded on January 02, 2017. This study covered pain episodes recorded by users between September 13, 2011 and January 02, 2017.

### Participants and Measures

The primary dataset included 544,425 records from 24,816 users. From these users, we selected 18,324 users who had recorded at least 2 pain episodes. The total number of data points from these 18,324 selected users was 537,853. We excluded users with only 1 pain record as we considered them as having only engaged with the app through a single use. In addition, the objective of our research was to highlight differences between users with varying degrees of engagement, whereas including those with a single-use would sway the analysis towards comparing engaged versus single-use instead.

We defined user engagement with the app using 2 aspects of usage: longevity and number of records. Longevity was calculated as the number of days between the first and the last pain record. The number of records was the total number of entries by a user in the database. For each user, we extracted 6 features from the database for the cluster-based analysis [15] (Textbox 1).

**Features extracted from the database for the cluster-based analysis.**

**Features**

Gender: The options for entering gender in the app are limited to either male or female. Users who did not include their gender information, or did not identify with either of the provided options, were coded as “not provided.” The percentages of male, female, and “not provided” genders in the set of selected users were 11.33% (2076/18,324), 49.90% (9144/18,324), and 38.77% (7104/18,324), respectively.

Age: The age (in years) recorded was the age of the user on the date of the first record and not as of the date of the analysis. Some users did not enter a date of birth. The age values for such users were not included in the analysis. Of the users, 57.48% (10,533/18,324) provided the age information.

Number of pain conditions: Users can select 1 or more pain conditions from a given list of 2500 different pain conditions. They can also add custom values to the pain conditions. In the present study, we included all pain conditions reported, including those added as custom values. Of the users, 57.68% (10,569/18,324) reported at least 1 pain condition.

Number of current medications: Users select their current and past medications from a standardized list of 1130 medications. In addition to instructions on how and when to take the medication, users can specify the brand and strength for each. If a medication or a brand is not found on the list, users can request to have it added. The present analysis included all medications that a user has indicated they are currently taking. Of the users, 36.96% (6773/18,324) reported taking 1 or more current medications.

Opioid use: For the purpose of the present study, a user of the app was coded as an opioid user if they self-reported taking at least 1 current medication containing bevorphanol, buprenorphine, butorphanol, codeine, fentanyl, hydromorphone, meperidine, methadone, morphine, oxycodone, oxymorphone, tapentadol, or tramadol. Of the users who reported taking 1 or more current medications, 41.21% (2791/6773) were coded as opioid users.

Pain severity rating: A user of the app enters a pain severity rating (0 to 10) for each pain record he/she creates. We first calculated mean daily pain severity ratings for each of the days a user created at least 1 pain record and then calculated the mean of the daily means for an average pain rating for each user. All users were assigned to the “Mild” (average pain rating less than 4/10), “Moderate” (average pain rating between 4 and 7), or “Severe” (average pain rating greater than 7) [15] group based on the average pain ratings.

### Data Analysis

#### Clustering

We applied data clustering methods to distinguish between highly engaged users and users who do not regularly use the app. Clustering involved partitioning a set of objects, or members of a defined population, into 2 or more subgroups such that the members of 1 subgroup were similar to each other and dissimilar to members of the other subgroups. Each object or subgroup member was represented using one or more variables for the purpose of clustering. These variables were typically referred to as features or attributes. The similarity or dissimilarity between pairs of objects (or subgroup members) was measured as the distance between the feature vectors representing them.

The output of the clustering process was usually a set of clusters where each object was assigned membership in 1 of the clusters. We used the method known as k-means [16] as our primary data analytic approach to clustering users. Under the k-means clustering method, the number of clusters is set a priori to some constant k, and the dataset is partitioned into k clusters. In the initialization stage, the k means were selected at random. Each item in the dataset was assigned to the mean closest to it. In each subsequent iteration, for each cluster, the mean was calculated based on the current members of that cluster. Each data point was then re-assigned to the cluster whose mean was the closest. The iterative process stopped when the clusters did not change between iterations.

In our clustering experiment, since we were interested in user engagement, we used the 2 defining variables—longevity and number of records—as features of user engagement. We also added frequency (average number of records per day) as an extra feature to distinguish between users who had the same number of records over different periods of longevity. We transformed these 3 feature values using a logarithmic scale because the difference between small feature values of 2 users was more indicative of their different levels of engagement than a similar difference in large feature values.

We compared the k-means clustering solutions that produced different numbers of clusters to find the solution with the best fit to the data. We also compared the k-means clustering results with results obtained using Mclust [17], another clustering method. In Mclust, a maximum number of clusters, M, and a set of mixture models were initially chosen. For each of these models, hierarchical agglomerative clustering was applied to obtain an initial clustering for each possible number of clusters from 2 to M. Using these clusters for each model as the base clusters, expectation-maximization algorithm was applied to update cluster assignments of objects for the number of clusters from 2 to M. Finally, Bayesian information criterion was used to choose a clustering solution from different models and different numbers of clusters.

To compare the quality of the clustering solutions between different methods (k-means versus Mclust), we calculated the average silhouette width as a measurement of the fitness of the clustering process. For each object, the silhouette width measured how much more similar (based on a distance measure such as Euclidean distance) a data point is to the points in its own cluster than to points in a neighboring cluster. Higher average silhouette widths indicated tighter clusters where each cluster was well-separated from other clusters.

After choosing a clustering solution based on average silhouette width, we generated a profile for each cluster of users. A cluster’s profile contained the means of 3 variables (user's age, number of pain conditions, and the number of current medications) calculated from the members that belonged to that cluster. For each cluster, we also calculated distributions of genders and pain severity levels and percentage of opioid users.

We used R (version 3.3.1) [18] for data loading, pre-processing, clustering, and conducting statistical tests. Notably, the traditional way of handling a dataset as a data-frame in R was slow for analysis of larger datasets. As our dataset contained more than half-a-million pain records, we used the data.table package [19] which made loading, querying, sorting, etc, quicker than the default data-frame approach.

#### Characterizing the Clusters

Once we had determined that we had generated the clusters that best represented the dataset, as evaluated by pain experts, we conducted a chi-square test to evaluate the statistical significance of the association between gender and cluster. We then conducted Z tests to determine whether the proportion of males and females in each cluster differed significantly from what one would expect by chance. We then conducted an analysis of variance (ANOVA) using 3, 2-way independent samples on the 3 database features (age, number of current medications, and number of pain conditions) using cluster and gender as the between-subject factors. We conducted pairwise comparisons using the Tukey honest significant difference (HSD) method for each significant main effect of cluster or gender. We then conducted a Z test to evaluate whether the proportion of males and females using opioids in each cluster differed significantly from what one would expect by chance. Finally, we conducted chi-square tests to investigate the association between gender and pain severity groups (mild, moderate, and severe) in each engagement cluster. All statistical tests were conducted in R.

## Results

### Clustering the Users Based on Their Engagement With the App

**Figure 2:**
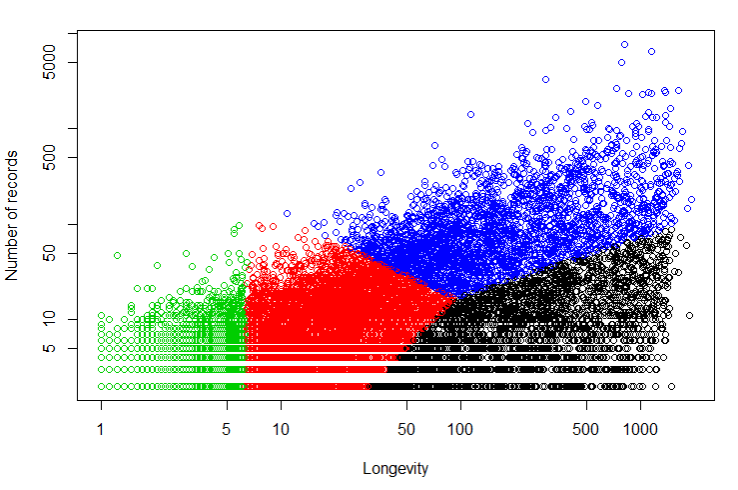
Clustering solution using 4 clusters.

The set of 18,324 users who had 2 or more pain records were clustered based on their level of engagement as measured by longevity, number of records, and frequency. We initially intended to divide users into the following natural groups based on their level of engagement: (1) low longevity, low number of records; (2) low longevity, high number of records; (3) high longevity, low number of records; and (4) high longevity, high number of records. The results from clustering the users into 4 groups are shown in Figure 2. The figure was plotted in the logarithmic scale of the 2 dimensions: longevity and number of records, where the 4 colors represented 4 different clusters. As described earlier, frequency (average number of records per day) was added as an additional variable during the clustering analysis to help emphasize differences between the various engagement clusters more relevant to the user base. The color of a cluster was assigned to all its member-objects (ie, the users who belonged to that cluster).

**Figure 3:**
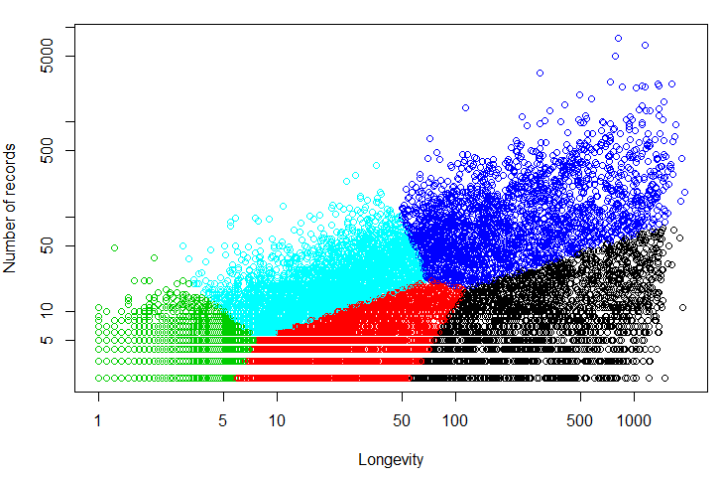
Clustering solution using 5 clusters where Blue is high longevity, high number of records; Black is high longevity, low number of records; Cyan is low longevity, high number of records; and Red and Green are low longevity, low number of records.

The Blue cluster represented users with high longevity and high number of records. Similarly, the users in the Black cluster generally had high longevity, but low number of records. However, the other 2 clusters (Red and Green) did not seem to align with the 2 other intended clusters characterized by (1) low longevity, high number of records; and (2) low longevity and low number of records. Instead, the Red and Green clusters appeared to differ at the low-end of longevity, but were similar in terms of representing low engagement. Hence, we conducted the clustering experiment again using 5 clusters (Figure 3).

The statistics derived from users of all 5 clusters are shown in Table 1. We discovered the following association between the clusters and the intended 4 groups of users based on the means of longevity and number of records as calculated from the users in a cluster: (1) Blue: high longevity, high number of records; (2) Black: high longevity, low number of records; (3) Cyan: low longevity, high number of records; and (4) Red and Green: low longevity, low number of records (Figure 3).

We also found that the average silhouette width was higher for the clustering results produced by k-means (0.20) than that produced by Mclust (0.02). Hence, we accepted the 5-cluster output of k-means, as shown in Figure 3, for further experiments in this study.

**Table 1:** Cluster characteristics according to the 5-cluster solution.

Cluster	Users, n	Longevity, n (days)			Records, n		
		Minimum	Maximum	Mean	Minimum	Maximum	Mean
Blue	2415	49	1906	321.8	18	7699	158.2
Black	2387	56	1865	418.5	2	76	12.6
Cyan	3640	3	67	21.5	6	34	22.7
Red	3467	5	109	30.1	2	21	6.1
Green	6415	1	7	2.6	2	47	3.4

### Cluster by Gender Profiles

**Figure 4:**
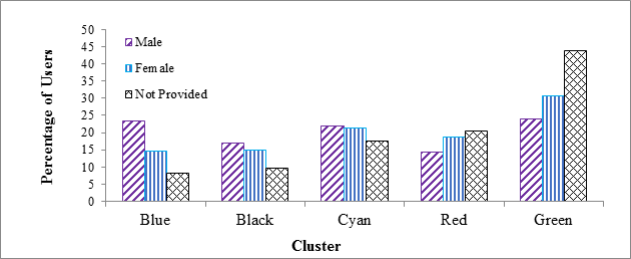
The distribution of users from each gender category across each of the 5 engagement clusters.

The distribution of users from the 3 categories of gender (male, female, not provided) across each of the 5 engagement clusters is shown in Figure 4.

A chi-square test was conducted to evaluate the association between 3 genders (male, female and not provided) and 5 clusters. The association was statistically significant (χ^2^_8_ = 761.24, *P* <.001 ).We then conducted Z-tests to evaluate whether the proportions of each pair of genders (male-female, male-not provided, female-not provided) differed significantly in each cluster. Pairwise between-gender differences were significant *(* all *P* ≤.05) for all clusters except the male-female difference in the Cyan cluster. Thus, the proportion of male users with apparent high longevity (Blue and Black clusters) was higher than the female users. On the other hand, for the low engagement clusters (Red and Green), the proportion of females was significantly higher than males. As noted above, only 11.33% (2076/18,324) of users in this study were male whereas 49.90% (9144/18,324) were female—with the remaining users not providing gender information. These data showed that once males registered, they were more likely to use the app for a longer period and with more consistency than females. It is notable that the proportion of the sample that did not provide a gender decreased significantly as user engagement increased from a high of 44% in the Green cluster to a low of 8% to 10% in the Blue and Black clusters, respectively. While knowledge of the genders of the individuals who chose not to provide gender data would be helpful in interpreting the present results, the finding that only 8% to 10% of the Blue and Black clusters did not provide their gender gave us more confidence that a greater proportion of males than females in the highly-engaged clusters was truly representative of the gender distribution and was not an artifact of the undeclared proportion.

Moreover, 68.37% (4857/7104) of these users in the “not provided” gender category did not enter their age and did not list any pain condition or current medications. On the other hand, this percentage was only 2.70% (56/2076) and 1.61% (147/9144) for males and females, respectively. Thus, comparing age, pain conditions, and medications between users who provided their gender information versus those who did not was not feasible. Hence, we excluded the users in the not provided gender category from the rest of the analysis.

To investigate the possible reasons behind the higher level of engagement of male users than female users, we calculated the mean age, mean number of pain conditions, and the mean number of current medications for both genders in each cluster.

#### Age

The mean age of the users from the 5 clusters is shown in Table 2. The results of an ANOVA revealed significant main effects of cluster (*F*_4,10192_= 24.09, *P*<.001) and gender (*F*_1,10192_ = 284.88, *P* <.001) but not the cluster x gender interaction effect (*F*_4,10192_ = 1.0, *P*=.41). Tukey HSD tests showed that the Blue and Black clusters each were significantly older than the 3 other clusters (all *P*<.001). In contrast, the Blue and Black clusters did not differ significantly (*P* =1.0). Thus, the average age of users with high longevity (Blue and Black clusters) was higher than that of the other groups of users. Overall, males were significantly older than females with mean ages of 42.32 (SD 12.01) and 37.55 (SD 10.56) years, respectively.

**Table 2:** Mean age of males and females in each cluster.

Cluster	Age, mean (SD)		
	All users	Males	Females
Blue	40.3 (10.9)^a^	44.1 (11.0)	39.1 (10.5)
Black	39.9 (11.1)^a^	42.9 (12.6)	39.2 (10.6)
Cyan	38.1 (11.1)^b^	41.9 (11.7)	37.2 (10.8)
Red	37.1 (11.4)^b^	40.5 (12.9)	36.6 (11.1)
Green	37.5 (11.2)^b^	41.8 (12.0)	36.8 (10.9)

^a^Cluster differed significantly by ANOVA (*P*<.001).

^b^Cluster differed significantly by ANOVA (*P*<.001).

#### Number of Pain Conditions

The mean number of self-reported pain conditions for the users in each cluster is presented in Table 3. More engaged users were more likely to self-report a higher number of pain conditions than less engaged users. The results of an ANOVA revealed significant main effects of cluster (*F*_4,9180_ = 41.28, *P*<.001) and gender (*F*_1,9180_ = 19.92, *P*<.001) but not the cluster x gender interaction effect (*F*_4,9180_ = 0.37, *P*=.83). Pairwise comparisons using post-hoc Tukey HSD tests indicated that the difference between the means for the Blue-Black, Black-Cyan, Cyan-Red, and Red-Green clusters were not statistically significant (all *P* ≥ .07). Across clusters, females reported more pain conditions than did males with mean values of 3.66 (SD 4.02) and 3.18 (SD 3.44), respectively.

#### Number of Current Medications

The mean number of current medications for users who reported taking at least 1 medication is shown in Table 4. The results of an ANOVA revealed significant main effects of cluster (F_4,5408_ = 58.67, *P*<.001) and gender (F_1,5408_ = 4.33, *P*=.04) but not the cluster x gender interaction effect (F_4,5408_ = 1.59, *P*=.17). Follow-up pairwise comparisons using post-hoc Tukey HSD tests revealed that the difference between the means of the Black-Cyan and Red-Green clusters were not significant (*P*>.99), whereas the difference between the Blue and each of the other clusters was significant (all *P*<.001). Thus, more engaged users reported taking more medications than did less engaged users. Moreover, females reported taking a greater number of pain medications than males, with mean values of 3.91 (SD 3.30) and 3.68 (SD 3.32), respectively.

**Table 3:** Mean number of pain conditions for males and females in each cluster.

Cluster	Pain conditions, mean (SD)		
	All users	Male	Female
Blue	4.3 (4.8)^a^	4.1 (4.5)	4.6 (4.8)
Black	3.8 (3.7)^a,b^	3.2 (3.0)	4.1 (3.9)
Cyan	3.4 (4.0)^b,c^	3.1 (3.3)	3.7 (4.4)
Red	3.1 (3.3)^c,d^	2.8 (2.5)	3.2 (3.4)
Green	3.0 (3.5)^d^	2.6 (2.7)	3.2 (3.6)

^a^Cluster differed significantly by ANOVA post-hoc Tukey HSD tests (*P*<.05).

^b^Cluster differed significantly by ANOVA post-hoc Tukey HSD tests (*P*<.05).

^c^Cluster differed significantly by ANOVA post-hoc Tukey HSD tests (*P*<.05).

^d^Cluster differed significantly by ANOVA post-hoc Tukey HSD tests (*P*<.05).

**Table 4:** Mean number of current mediations for males and females in each cluster.

Cluster	Mean Number of Current Medications (SD)		
	All users	Male	Female
Blue	4.6 (4.0)^a^	4.7 (4.4)	5.0 (4.0)
Black	3.7 (3.1)^b^	3.1 (2.4)	4.1 (3.4)
Cyan	3.6 (3.0)^b^	3.4 (2.6)	4.0 (3.2)
Red	3.0 (2.6)^c^	3.1 (2.5)	3.3 (2.7)
Green	2.8 (2.4)^c^	2.8 (2.2)	3.2 (2.7)

^a^Cluster differed significantly by ANOVA post-hoc Tukey HSD tests (*P*<.001).

^b^Cluster differed significantly by ANOVA post-hoc Tukey HSD tests (*P*<.001).

^c^Cluster differed significantly by ANOVA post-hoc Tukey HSD tests (*P*<.001).

### Opioid Use

The number and percentage of males and females within each cluster reporting the current use of an opioid are shown in Table 5. The percentage of males taking an opioid was significantly greater (all *P* ≤ .05) than that of females for all clusters except the Red cluster where the percentages did not differ.

### Pain Severity Rating

The number of male and female users in 3 pain severity groups within 5 clusters is shown in Table 6. Chi-square test of independence revealed that pain severity and gender were independent of each other in the Black and Cyan clusters (all *P* ≥ .40). We conducted follow-up chi-square tests between gender and pairs of pain severity groups for the Blue, Red, and Green clusters. In the Blue cluster, mild-severe (χ^2^_1_= 11.18, *P*=.008) and mild-moderate (χ^2^_1_ = 9.65, *P*=.002) pairs had a statistically significant association with the male and female genders. The association between the mild-moderate pair and gender was significant in the Red (χ^2^_1_ = 8.09, *P*=.004) and Green (χ^2^_1_= 12.76, *P*<.001) clusters. These findings indicated that across the Blue, Red, and Green clusters, the proportion of males with mild pain was significantly higher than that of females.

**Table 5:** Number and percentage of each gender reporting current opioid use within each cluster where the percentages for each gender were calculated using a denominator that comprised the number of that gender taking at least 1 current medication in a cluster.

Cluster	Males taking an opioid, n (%)	Females taking an opioid, n (%)
Blue, males (N=342) and females (N=950)	183 (53.5%)	450 (47.4%)
Black, males (N=187) and females (N=699)	102 (54.5%)	291 (41.6%)
Cyan, males (N=221) and females (N=1074)	114 (51.5%)	432 (40.22%)
Red, males (N=94) and females (N=683)	42 (44.7%)	250 (36.6%)
Green, males (N=155) and females (N=1013)	75 (48.4%)	379 (37.41%)

**Table 6:** Number and percentage of male and female users by pain severity groups within each cluster.

Cluster	Male users, n (%)			Female users, n (%)		
	Mild pain	Moderate pain	Severe pain	Mild pain	Moderate pain	Severe pain
Blue^a^, males (N=485) and females (N=1340)	108 (22.0%)	266 (54.8%)	111 (22.9%)	205 (15.30%)	775 (57.84%)	360 (26.87%)
Black, males (N=348) and females (N=1355)	59 (16.9%)	210 (60.3%)	79 (22.7%)	212 (15.65%)	831 (61.33%)	312 (23.02%)
Cyan, males (N=452) and females (N=1937)	93 (20.6%)	256 (56.6%)	103 (22.8%)	346 (17.86%)	1139 (58.80%)	452 (23.33%)
Red^b^, males (N=295) and females (N=1709)	70 (23.7%)	159 (53.9%)	66 (22.4%)	292 (17.09%)	1037 (60.66%)	380 (22.23%)
Green^b^, males (N=496) and females (N=2803)	153 (30.8%)	238 (48.0%)	105 (21.2%)	661 (23.58%)	1540 (54.94%)	602 (21.48%)

^a^Mild-severe (*P*=.008) and mild-moderate (*P*=.002) pairs had a statistically significant association with the male and female genders.

^b^Mild-moderate pair and gender were significantly associated (*P* ≤ .004).

## Discussion

### Principal Findings

Commercially-available apps to track and record pain have proliferated to the point where consumers and healthcare providers alike face a bewildering array with little data to use in making an informed decision regarding options. The pain literature regarding mHealth apps typically focuses on app validation, clinical efficacy, or engagement, but no other study has applied data mining techniques to a large user data base of chronic pain sufferers. With more than 250 commercially-available apps to choose from users have little reliable information to turn to when looking for the best app for their needs. The results of the present study provided an in-depth look at the user base of the Manage My Pain app and described factors associated with high user engagement.

The main objective of the present study was to use data mining (clustering) methods to analyze engagement patterns from users of Manage My Pain according to several key variables that defined the user base. Specifically, we categorized users based on their gender and level of engagement with the app. The results of the present study were novel in several respects. For one, to the best of our knowledge, this was the first application of clustering methods to describe patterns of use of, and engagement with, a pain-monitoring app among a large number of everyday users who report chronic pain. We used a sample of 18,324 users who recorded at least 2 pain episodes and together generated more than 500,000 records. Using the k-means clustering approach, the users were classified into 5 distinct clusters that differed maximally in user engagement, derived from their frequency and longevity of use. The Blue and Black clusters comprised individuals with high longevity and a large and small number of records, respectively, whereas the Red and Green clusters comprised individuals with low longevity and a relatively small number of records. The Cyan cluster represented individuals with low longevity and relatively large number of records. We then examined the differences among the 5 clusters with respect to gender, age, number of pain conditions, number of pain medications, opioid use, and pain severity rating.

The most highly engaged clusters (Blue and Black), which were distinguished by frequency of app use but not longevity, differed only in number of pain medications which were greater in the Blue than Black cluster. Otherwise, these clusters were similar in terms of relative gender composition, age, number of pain conditions reported, and proportion using opioids. Together these most engaged clusters comprised 4802 individuals who used the app for an average of 1 year. Compared with the less engaged clusters (Red and Green), the more engaged clusters (1) were, relative to the total number of males and females, more likely to be male; (2) were significantly older; (3) reported a significantly greater number of pain conditions; and (4) were more likely to be opioid users.

Another potentially important result pertained to the distribution of gender within the 5 clusters. The proportion of males and females in each cluster, except Cyan, differed significantly from what would be expected by chance alone. However, the proportions differed markedly based on user engagement. Among the most engaged clusters of users (Blue and Black), the proportion of the total sample of male users was significantly greater than that of females. In contrast, the opposite was true for the least engaged clusters of users (Green and Red) where the proportion of the total sample of female users was significantly greater than that of males. Although only 11.33% (2076/18,324) of users were male and 49.90% (9144/18,324) females, the data indicated that once males registered, they were more likely to use the app for a longer duration and more consistently than females. The greater proportion of males than females in the highly-engaged cluster was interesting because males typically are less actively engaged in their own healthcare than females [20-22]. For instance, females visit primary care providers more frequently than males [21,22] and adhere more to physician recommendations [23]. In a study of 3.7 million patients registered with primary care physicians in the United Kingdom, the rate of consultation for males was 32% lower than it was for females, with the greatest gender differences seen in patients between the ages of 16 and 60 years [22]. Healthcare-seeking behaviors are also more frequent among females than males who have sustained whiplash injuries and who we would reasonably expect to have pain [24].

Gender differences in the use and uptake of mHealth technology may help to explain the present results [25,26]. In contrast to the greater healthcare-seeking behaviors in females, males are more likely than females to adopt mHealth technology [25]. Moreover, whereas males tend to find mHealth apps helpful in averting a health problem and in benefiting from them, this is not the case for women [26]. We suggest that the greater proportion of males than females in the highly-engaged cluster may be related to the mobile-based medium through which the pain-related information is self-monitored and recorded. Use of a mobile pain app may be relatively more attractive to highly-engaged males than highly-engaged females and may be one way to increase male uptake of healthcare behaviors in general.

The relationship between pain severity and gender within clusters indicated that overall, for the Blue, Red, and Green clusters, the proportion of males with mild versus moderate pain was significantly lower than would be expected by chance alone. In the context of the published literature, they are not surprising since across pain conditions males tend to report lower levels of pain than females [27]. It is interesting to note, however, that this pattern was also true for males with mild versus severe pain in the Blue cluster, which, as previously noted, was the most engaged cluster being both high in longevity and high in number of records. Moreover, this was one of the clusters in which the proportion of the total sample of male users was significantly greater than that of females indicating that once males registered, they were more likely to use the app for a longer duration and more consistently than females. The pain severity by gender association in the Blue cluster suggested that the high engagement by males in this cluster did not appear to be driven by pain severity which may, in part, be the case for females.

Consistent with the published literature showing that women are more likely than men to engage in polypharmacy [28,29], the present results indicated that females in all clusters reported taking a greater number of pain medications than males. Likewise, the percentage of males who reported using opioids was significantly greater than that of females in all but the Red cluster. These data are consistent with prior reports where it was generally found that the frequency of opioid use among males with chronic pain was greater than that of females [30]. Results of a treatment study [31] showed that prior to treatment, fewer females than males were using opioids and females were younger than males. This pattern appears consistent with a recent study [30] which showed that although frequency of opioid use among males with chronic pain was greater than that of females, overall, frequency of use tended to decrease with increasing age. For example, 81% of females with chronic pain aged 25 to 44 years reported using opioids whereas 76.5% of males aged 45 to 64 years reported using opioids. Thus, the present results may also reflect the fact that the highly-engaged males were older than highly-engaged females. These results are of interest and importance given the recent “opioid epidemic” that is the focus of increasing concern among patients with chronic, non-cancer pain, healthcare providers, regulators, and governments [32,33]. By tracking opioid use and pain severity over the next several years we will be in a position to provide important Manage My Pain user data on the extent to which the new opioid prescribing guidelines in the United States [34] and Canada [35] are associated with changes in these parameters.

A mismatch was noted by de la Vega and Miró [9] between the commercial sector and the scientific community in terms of their respective approaches to app development and evaluation. Of the 34 pain-related apps that were evaluated in the scientific literature, not one was available in any of the major online app stores. In contrast, of the more than 280 commercially available pain-related apps not one was the topic of a scientific publication. The present collaboration between the developers of Manage My Pain, scientists studying pain, and experts in data mining, was an attempt to address this mismatch and initiate a novel direction in pain research. By evaluating trends in how consumers engage with and use commercially-available apps to monitor and track pain we can begin to make inroads in understanding the motivations of populations that have been traditionally more difficult to engage (eg, males suffering with multiple pain conditions).

### Conclusion

This is the first study to use data mining (clustering) methods to analyze data from the mobile pain app Manage My Pain, according to several key variables that defined the user base. To better understand who uses the mobile pain app, Manage My Pain, clustering methods were applied to a sample of 18,324 users who recorded at least 2 pain episodes and collectively entered 537,853 records into the app. Users were grouped into 5 clusters according to their engagement patterns. Of the clusters, 2 were identified as representing high user engagement based on longevity and frequency of app use. All 5 clusters were first characterized by gender and then by age, number of pain conditions, number of current medications, opioid use, and pain severity rating. Although most users of the app reported being female, the cluster analysis indicated that male users were more likely to be highly engaged in the app. Clusters of highly-engaged users differed from the other clusters in terms of the relative composition of males and females, with a greater proportion of males than females in the former than the latter clusters. In addition, users in the most engaged clusters self-reported a higher number of pain conditions, a higher number of current medications, and a higher incidence of opioid usage. We suggest that use of a mobile pain app may be relatively more attractive to highly-engaged males than highly-engaged females, and to those with relatively more complex chronic pain situations. A mobile pain app, such as Manage My Pain, may be one way to increase uptake of healthcare behaviors in general for both males and people with complex chronic pain situations.

## Abbreviations

ANOVA: analysis of variance
HSD: honest significant difference
mHealth: mobile health
